# Addition of Silver Nanoparticles to Composite Edible Films and Coatings to Enhance Their Antimicrobial Activity and Application to Cherry Preservation

**DOI:** 10.3390/foods12234295

**Published:** 2023-11-28

**Authors:** Angelos-Panagiotis Bizymis, Styliani Kalantzi, Diomi Mamma, Constantina Tzia

**Affiliations:** 1 Laboratory of Food Chemistry and Technology, School of Chemical Engineering, National Technical University of Athens, 5 Iroon Polytechniou St., 15780 Zografou, Athens, Greece; apbizymis@yahoo.gr; 2 Biotechnology Laboratory, School of Chemical Engineering, National Technical University of Athens, 5 Iroon Polytechniou St., 15780 Zografou, Athens, Greece; stykalan@chemeng.ntua.gr (S.K.); dmamma@chemeng.ntua.gr (D.M.)

**Keywords:** silver nanoparticles, antimicrobial activity, composite edible films, food packaging, cherries

## Abstract

The aim of this study was to examine the potential enhancement of the antimicrobial activity of edible films, composed of (i) chitosan (CH), cellulose nanocrystals (CNC) and beta-cyclodextrin (CD) (50%-37.5%-12.5%) and (ii) hydroxypropyl methylcellulose (HPMC), cellulose nanocrystals (CNC) and beta-cyclodextrin (CD) (50%-37.5%-12.5%), with silver nanoparticle (AgNP) incorporationat levels 5, 10 and 15% *v*/*v*. According to the results, the AgNP addition led to very high antimicrobial activity of both films, reducing by more than 96% the microbial growth of the Gram-negative bacterium *Escherichia coli* (*E. coli*) in all cases. On the other hand, by adding AgNPs to films, their thickness as well as oxygen and water vapor permeability decreased, while their transparency increased. Furthermore, the contribution of these specific edible films to preserve cherries under cold storage was investigated. All edible coatings resulted in an improvement of the fruit properties under consideration, and especially the color difference, hardness and total microbial load.

## 1. Introduction

In recent years, consumers are increasingly looking for fresh, natural and nutritious food products such as fruits and vegetables. However, various microorganisms such as bacteria and fungi cause microbial spoilage in these products, reducing their shelf life and increasing the risk of foodborne illness [1]. Contamination of foods also causes loss in their texture, color and nutritional value, while allowing for the growth of pathogenic microorganisms, and degrades the total quality of the foods, making them unfit for consumption [1,2,3]. Especially in fruits and vegetables, the high decay rates due to nutrient loss and contamination by microorganisms result in extremely large losses worldwide every year [4,5].

As spoilage usually starts from the surface of food, proper surface treatment and packaging are required to prevent it [6,7]. Thus, new packaging methods are being explored to meet the growing need for safe and high quality foods [2,8]. Antimicrobial packaging, in particular, aims to delay or even inhibit the rate of spoilage and occurrence of pathogens [9,10]. In this context, edible films and coatings can serve as a type of antimicrobial packaging, as they provide a barrier to the food surface that is semi-permeable and blocks external sources of microorganisms. They can also help inhibit processes such as gas exchange, respiration rates, moisture loss and oxidative reactions, thus increasing the shelf life of products. In addition, they may contain additional antimicrobial substances, flavoring and coloring agents, growth regulators and antioxidants [3,4,11,12,13,14].

Silver is a material with a very high antimicrobial activity, much higher than that of other metals. In particular, the antibacterial mechanism of silver is related to its interaction with the thiol groups of protein molecules, thus binding the bacterial cell membrane and preventing the copying of DNA molecules [15,16,17].

In recent years, in addition to silver, silver nanoparticles (AgNPs) have also been studied for their antimicrobial properties. Actually, the use of nanomaterials is generally increasing in active food packaging. In particular, metal oxides such as AgNPs can help to enhance the mechanical and barrier properties of biodegradable films. Moreover, as they present a high surface-to-volume ratio, leading to increased surface activity, AgNPs can achieve strong contacts with microorganisms, leading to an enhanced antimicrobial effect [18,19,20,21,22].

However, there is not enough evidence as to whether nanomaterials are safer or more dangerous compared to conventional ones. There are still no specific regulations for certain migration limits for nanomaterials [12,20,23,24]. Nevertheless, AgNPs can be considered non-toxic and safer compared to carcinogenic fungicides, if they are combined with non-toxic materials [24,25]. Indeed, 125 mg/kg (ppm) has been suggested as a safe upper limit for AgNP content [26,27].

The present study examines the incorporation of AgNPs into two types of novel composite edible films introduced in previous studies, showing remarkably improved properties (e.g., high oxygen and water vapor barrier and high mechanical structure) [28,29]. More specifically, the initial composition of the films included: (i) chitosan (CH), cellulose nanocrystals (CNC) and beta-cyclodextrin (CD) and (ii) hydroxypropyl methylcellulose (HPMC), cellulose nanocrystals (CNC) and beta-cyclodextrin (CD). In this study, AgNPs were incorporated in alternative proportions in CH-CNC-CD and HPMC-CNC-CD solutions, in order to examine, on the one hand, the antimicrobial activity and the induced changes in the behavior of the final edible films and, on the other hand, the results of their application to the preservation of cherries.

In conclusion, whereas most studies focus specifically on final coated edible products [30,31,32,33,34,35,36,37], the present study examines not only the coated fresh fruits (cherries), but, before that, the edible films themselves in innovative compositions.

## 2. Materials and Methods

### 2.1. Materials

For the preparation of the edible films, the following materials were used: high molecular weight CH (Deacetylated chitin, ≥75% deacetylated, molecular weight 310,000–375,000 Da, Poly[D-glucosamine]) from Sigma-Aldrich (St. Louis, MO, USA), acetic acid (glacial 99–100% a.r.) from Chem-Lab NV (Zedelgem, Belgium), HPMC (methoxyl 28–30% and hydroxypropyl 7–12%) from Alfa Aesar (Ward Hill, Haverhill, MA, USA), CNC from CelluForce (Montreal, QC, Canada), CD from Acros Organics (Geel, Belgium) and AgNPs (50 ppm) from Full Health (Athens, Greece). The CNC specifications were: molecular formula: [(C_6_O_5_H_10_)_22–28_SO_3_Na]_4–6_, specific surface area: 400 m^2^/g, molecular weight (g/mol): 14,700–27,850, particle size: 1–50 μm, particle diameter (crystallite): 2.3–4.5 nm (by AFM), particle length (crystallite): 44–108 nm (by AFM), crystalline fraction: 0.88 (by XRD) and crystallite density: 1.5 g/cm^3^. The CD specifications were: molecular formula: C_42_H_70_O_35_, molecular weight (g/mol): 1134.99, solubility in water: soluble, specific rotation condition: +150.00 (25 °C c = 1.5, in water) and purity: 98%. The cherries (Early Lory Cherry variety) were obtained from the company Bourakis Super Fruits (Thessaloniki, Greece).

### 2.2. Preparation of Edible Films

Initially, solutions of CH-CNC-CD and HPMC-CNC-CD, in 50%-37.5%-12.5% proportions, were prepared, as described in Bizymis et al. [28,29]. Amounts of the 50 ppm AgNP solution were then added, at ratios of 0, 5, 10 and 15% *v*/*v*. Thereafter, the final solutions (with AgNP concentrations of 0, 2.5, 5.0 and 7.5 ppm, respectively) were homogenized, using a high-speed homogenizer (CAT Unidrive 1000, CAT Scientific, Paso Robles, CA, USA) for 10 min at a speed of 8000 rpm. All resulting solutions were degassed at 30 °C for 15 min, using an Elmasonic Ultrasonic device S30H (Elma Schmidbauer GmbH, Singen, Germany) (280 W/60 Hz). Subsequently, 20 mL aliquots were taken from the final solutions, placed in 9 cm Petri dishes and allowed to dry for 24 h at 50 °C in a vacuum oven. Finally, the dried films were kept at room temperature and humidity.

### 2.3. Measurements in the Edible Films

#### 2.3.1. Thickness

To determine the film thickness (mm), a stack of 7 films per type was taken. Following this, measurements were made at 5 different points of each film sample, using a hand-held micrometer (Kalibr Instrument Plant, Moscow, Russia), having a measurement capability of 0.01 mm and a tolerance (permissible error) of ±0.004 mm. Consequently, 35 measurements for each type of film were performed. Finally, for each type of film, the average value of the 35 measurements was estimated.

#### 2.3.2. Mechanical Properties

The evaluation of the mechanical properties was performed according to the ASTM D882-10 [38] standard. A TA-XT2i Texture Analyzer (Stable Micro Systems, Godalming, UK) with a 5 mm cylindrical probe was used for the Texture Analysis, as in Bizymis et al. [28]. The measurements were taken as described in Bizymis et al. [28].

#### 2.3.3. Oxygen Permeability

A liquid analysis system was used to measure the oxygen permeability, with iodometry application. The system was based on the ASTM D3985-05 [39] standard. The procedure was performed as described in Bizymis et al. [28] and Vogel [40]. Briefly, oxygen was allowed to pass through each film and was transferred to the liquid analysis system by means of nitrogen movement. A suitable metal cup was used to supply oxygen that sealed one side of the film, while for nitrogen supply a similar metal cup sealed the other side.

The mass (*m*) of the permeated O_2_ was derived from the volume of the standard thiosulphate solution consumed during titration, in the context of iodometry in the liquid analysis system. Specifically, 1 mL of the standard thiosulphate solution corresponded to 1 mg of O_2_.

The entire procedure and the applied equations are described in detail in Bizymis et al. [28].

The Oxygen Permeability (*OP*) of the films was calculated as follows:

(1) $$OP=\frac{m\cdot d\;}{A\cdot t\cdot \Delta P}$$

where *m* is the mass of O_2_ which passed through the film in a time interval *t*, *d* is the film thickness, *A* is the film area and Δ*P* is the O_2_ pressure difference between the two sides of the film.

#### 2.3.4. Water Vapor Permeability

For the measurement of water vapor permeability, the method of Bertuzzi et al. [41] and Bizymis et al. [28] was applied. Briefly, distilled water was poured into glass cups, edible films were sealed the cups and finally the cups were put into a desiccator containing saturated sodium chloride (NaCl) solution with a relative humidity of 75%. Different levels of relative humidity on each side of a film was applied to achieve the movement of water vapor, tending to balance the humidity of the two sides. In the desiccator, the temperature was 40 °C and no ventilation was applied. Periodic measurements of the weight changes of the cups were then taken during 24 h. Throughout the process, the inside of the desiccator was not affected by the external environment, except for six times, when it had to be opened to take measurements of the weight changes of the cups. The period of 24 h proved to be sufficient time to obtain comparative water vapor permeability results, so no extension of the duration was deemed necessary.

Using linear regression, the Δ*m*/Δ*t* ratio was then calculated.

The Water Vapor Permeability (*WVP*) of the films was calculated as follows:

(2) $$WVP=\frac{\Delta m\;}{\Delta t\;}\cdot \frac{l\;}{A\cdot \Delta P\;}$$

where Δ*m* is the amount of water vapor passing through the film over a time interval Δ*t*, *l* is the film thickness, *A* is the film area and Δ*P* is the vapor pressure difference across the film.

#### 2.3.5. Color

A CR-200 Colorimeter (Konica Minolta, Basildon, UK) was used to determine the color of the films. For the evaluation, the color parameters *L* (Luminosity), *a* (Red–Green) and *b* (Yellow–Blue) of the Cielab scale were applied. As a standard, between the possible options of a white plate and black trap cup, the white plate option was chosen (*L*_0_, *a*_0_ and *b*_0_).

The calculations were made as follows [42,43]:

Color difference:

(3) $$\Delta E=\sqrt{{\left(\Delta L\right)}^{2}+{\left(\Delta a\right)}^{2}+{\left(\Delta b\right)}^{2}}$$

Whiteness index:

(4) $$WI=100-\sqrt{{\left(100-L\right)}^{2}+a^{2}+b^{2}}\;$$

Chrome:

(5) $$C^{*}=\sqrt{a^{2}+b^{2}}$$

where Δ*L* = *L* − *L*_0_, Δ*a* = *a* − *a*_0_ and Δ*b* = *b* − *b*_0_.

#### 2.3.6. Antimicrobial Activity

The antimicrobial activity of the edible films was assessed against the Gram-negative bacterium *Escherichia coli* (*E. coli*), according to the ISO 22196:2011 [44] standard. The bacterium *E. coli* was chosen as a representative Gram-negative bacterium, which often is reported to contaminate water and poorly processed foods (e.g., vegetables, unpasteurized milk, dairy products, juices, sausages), and some of its strains can endanger public health, even at the level of epidemic outbreaks. Film samples were cut to a size of 5 cm × 5 cm, sterilized with a UV lamp for 30 min on each side and placed into sterile Petri dishes. *E. coli* was grown on Nutrient Broth (NB) liquid medium in an orbital shaker operating at 200 rpm and 30 °C for 16 to 20 h.

An amount of 0.4 mL of *E. coli* culture, at a cell density of 10^6^ cells/mL, was spread onto the surface of the 5 cm × 5 cm films. A piece of sterilized polypropylene film (4 cm × 4 cm) was then placed on the films and the inoculated samples were incubated at 30 °C for 24 h. After incubation, each film sample was treated with 10 mL of soybean casein digestion broth with lecithin and polyoxyethylene sorbitan monooleate (SCDLP), to recover the bacteria. After that, 10-fold serial dilutions of the SCDLP in phosphate-buffered physiological saline were performed. Then, 100 μL of the undiluted SCDLP containing *E. coli* cells recovered from the films and the serial dilutions were spread on Petri dishes containing plate count agar (yeast extract 2.5 g/L; tryptone 5.0 g/L; glucose 1.0 g/L and agar powder 15.0 g/L). After incubation at 30 °C for 24 h, the number of *E. coli* colonies in the Petri dishes was counted.

The above procedure was also performed on blank samples without AgNPs.

Antimicrobial activity (*R*) and % reduction of microbial growth were calculated as follows:

(6) $$R=U_{t}-A_{t}$$

(7) $$\%\;Reduction\;of\;Microbial\;Growth=\frac{Colonies_{blank\;sample}-Colonies_{sample\;with\;AgNPs}}{Colonies_{blank\;sample}}\cdot 100\;$$

where *U_t_* is the average of the common logarithm of viable bacteria per cm^2^, recovered from the samples without AgNPs after 24 h, and *A_t_* is the average of the common logarithm of viable bacteria per cm^2^, recovered from the samples with AgNPs after 24 h.

According to Flak et al. [45], Scuri et al. [46] and JIS L 1902:2015 [47] the antimicrobial activity is classified as follows:

i. *R* ≤ 0.5 no antimicrobial activity (up to 68.4% reduction of microbial growth);
ii. 0.5 < *R* ≤ 1.0 slight antimicrobial activity (over 68.4% and up to 90% reduction of microbial growth);
iii. 1.0 < *R* ≤ 2.0 medium antimicrobial activity (over 90% and up to 99% reduction of microbial growth);
iv. 2.0 < *R* ≤ 3.0 good antimicrobial activity (over 99% and up to 99.9% reduction of microbial growth);
v. *R* > 3.0 very good antimicrobial activity (over 99.9% reduction of microbial growth).

### 2.4. Preparation of the Samples of the Cherries

First the CH-CNC-CD and HPMC-CNC-CD solutions were prepared with incorporated AgNPs at levels of 0, 5 and 15% *v*/*v*, as described in Section 2.2. Next, the samples of cherries were placed in the solutions for 3 min and then allowed to dry for 30 min at room temperature. After drying, all samples were placed in PET/AL/PE (polyethylene terephthalate/aluminum foil/polyethylene) material bags (5 in each bag) and kept at 0 °C, which were considered as the appropriate conditions for the specific type and duration of the experiments. Control samples without edible films were also stored in the same way. In particular, the temperature was chosen to be low, to ensure that all samples would remain in a relatively good condition for the 14 days of storage, so that an effective comparison between all samples was possible.

### 2.5. Measurements in the Cherries

#### 2.5.1. Weight Loss

The weight of individual samples was measured right after treatment (storage time 0 day) and on different subsequent sampling dates. Weight loss (*WL*) was calculated as follows:

(8) $$WL=\frac{m_{0}-m_{t}}{m_{0}}\cdot 100$$

where *m*_0_ was the initial weight of the samples at storage time 0 day and *m_t_* was the weight of the samples on the day of sampling (3rd, 7th, 10th and 14th days).

#### 2.5.2. Color Difference

The calculation of color difference (ΔE) of the cherry samples was performed as in 2.3.5. with *L*_0_, *a*_0_ and *b*_0_ being the parameters of the control samples at storage time 0 day.

#### 2.5.3. Hardness

For the measurement of hardness, the TA-XT2i Texture Analyzer (Stable Micro Systems, Godalming, UK) with a cutting blade was used. The penetration depth and the crosshead speed were set to 5 mm and 1 mm/s, respectively. The Texture Analysis software provided the curves of force (N) versus deformation (mm). The hardness corresponded to the maximum force (N).

#### 2.5.4. Microbiological Analysis

For the measurement of the total microbial load, 5 g pieces were cut with a sterile knife from the cherries out of each bag and were homogenized with 45 g of sterile Ringer serum in a sterile bag using a Stomacher (BagMixer^®^ Interscience, Charles de Gaulle, France). Thereafter, serial dilutions were carried out with use of plate count agar as in Section 2.3.6. The samples were prepared in triplicate. Based on the ISO 22196:2011 [44] standard, for each sample, the dilution which gave a count of 30–300 colony forming units (CFU) was preferred for consideration. In cases where less than 30 colonies were counted in all dilutions of the same sample, the first solution with visible colonies was considered. In all cases, at least one dilution with less than 300 colonies was found. All Petri dishes were incubated for 3 days at 25 °C.

### 2.6. Statistical Analysis

Statistica 13.0 software (StatSoft, Inc., Tulsa, OK, USA) was used for statistical data processing. The ANOVA variance analysis was applied to evaluate each experimental factor. Significant differences were considered at the *p* < 0.05 level. Where applicable, Duncan’s multiple range test was used to indicate different levels between each set of means.

## 3. Results and Discussion

In the current study, the effect of incorporating AgNPs into CH-CNC-CD and HPMC-CNC-CD edible films (in proportions of 0, 5, 10 and 15% *v*/*v* for each case) was examined, especially to investigate whether AgNPs can provide antimicrobial activity to these films and at what levels. Subsequently, the effect of the specific films (with AgNP content in proportions of 0, 5 and 15% *v*/*v*) on the preservation of cherries was studied, by controlling the change of their appearance and of their structural and microbial indicators during the storage time. The parameters examined for the films and cherry samples are presented as follows.

### 3.1. Characterization of the Formed Edible Films

#### 3.1.1. Thickness and Mechanical Properties

The results for the thickness and mechanical properties of the film samples are presented in Table 1. In particular, the maximum breaking force (F), the elastic modulus, the percent of elongation at break (ε) and the breaking stress (σ) were measured.

According to the results in Table 1, both types of films (CH-CNC-CD and HPMC-CNC-CD) before incorporating AgNPs were quite thin. With the addition of AgNPs, the thickness of both types of films further reduced, despite an increase in solids content. This result contrasts with other researches, such as Vieira et al. [25], which showed an increase in the film thickness after the addition of AgNPs. However, the present decrease in film thickness can probably be attributed to the small size of the AgNPs that facilitates their fine dispersion into the polymer matrices, leading to more homogenous films [48]. In addition, the thickness of HPMC composite films was slightly lower (*p* < 0.05) than that of CH ones (Table 1). It must be mentioned that edible films with a thickness of less than 0.25 mm are characterized as thin [49]. The standard deviation value for almost all thickness measurements was zero due to the capability of the instrument used. A higher resolution instrument would possibly detect differences, however, as the measurements were well below the aforementioned limit of 0.25 mm, two decimal places of accuracy was deemed sufficient.

Regarding the mechanical properties, high values are important for edible films in order to protect food products and extend their shelf life. According to the results, all films were flexible and showed high mechanical strength, in agreement with similar studies [50,51,52,53,54,55]. However, the mechanical properties of CH-CNC-CD films were somewhat higher than those of HPMC-CNC-CD films.

The fact that both CH and HPMC films have high mechanical strength is also indicated by the experiments described in Bizymis et al. [28,29], who showed that 1% *w*/*v* HPMC plain films exhibited lower mechanical stability (F: 14.29 N and σ: 0.73 MPa) than 1% *w*/*v* CH plain films (F: 23.94 N and σ: 1.22 MPa). This can be explained due to the higher mechanical strength of the CH base material compared to the corresponding HPMC base material. In the same context, similar studies argue that CH forms edible films and coatings with advanced mechanical properties, while other commonly used materials, such as gelatin, form films with markedly lower performance [54,55].

Furthermore, as can be seen in Table 1, the addition of CNC or CD led to slightly inferior mechanical properties of both types of composite film, compared to the plain ones, indicating lower interactions between their molecules, while maintaining the CH films superior to the HPMC ones. The advantage of the CH-CNC-CD films compared to the HPMC-CNC-CD films can also be attributed to the higher mechanical strength of the CH base material compared to the corresponding HPMC base material.

Upon the incorporation of AgNPs, the mechanical properties were not significantly differentiated, thus suggesting that AgNPs played a less important role in these properties than HPMC, CH, CNC and CD. The fact that AgNPs did not have a negative impact on the mechanical properties is probably related to the ability of the nanocrystals to allow sufficient load transfer and stress distribution in the edible films. In any case, the addition of 15% *v*/*v* AgNPs to both types of films provided the best overall results for these properties.

#### 3.1.2. Barrier Properties

The barrier properties of the film samples (oxygen permeability and water vapor permeability) are presented in Table 2.

According to the results in Table 2, both types of films (CH-CNC-CD and HPMC-CNC-CD), before incorporating AgNPs, showed quite good levels of oxygen permeability (OP) and water vapor permeability (WVP) values, despite the tendency of CH and HPMC composite films to increase the relative humidity and form crosslinks with water [50,56,57,58]. Also, HPMC-CNC-CD films showed lower (*p* < 0.05) OP and WVP values compared to those of CH-CNC-CD.

Indeed, HPMC, is a polysaccharide that has a stable crystalline structure and forms edible films with very effective O_2_, CO_2_ and lipid barrier properties [59,60,61]. On the other hand, CH is also a polysaccharide, biodegradable, compatible with other substances and easy to adhere to various products. In addition, it also forms films that are good O_2_ and CO_2_ barriers [62,63,64].

Specifically, in experiments described in Bizymis et al. [28,29], 1% *w*/*v* CH plain films showed OP and WVP values of 2.99·10^−12^ g·s^−1^·Pa^−1^·m^−1^ and 2.20·10^−9^ g·s^−1^·Pa^−1^·m^−1^, respectively, whereas the same properties of 1% *w*/*v* HPMC plain films had values 1.98·10^−12^ g·s^−1^·Pa^−1^·m^−1^ and 1.84·10^−9^ g·s^−1^·Pa^−1^·m^−1^, respectively. Therefore, in the plain films, the HPMC base material was obviously advantageous over the CH.

Moreover, the barrier properties of both HPMC and CH can be further improved by adding suitable materials to the original solution. In particular, as shown in Table 2, both CNC and CD improved these properties, which can be explained as follows:

During the formation of edible films, the CNC nanomaterial forms strong hydrogen bonds with other polymers, with these interactions being more evident in water-soluble and hydrophilic polymers. This is related to the high crystallinity and low hygroscopicity of CNC. While the non-crystalline regions favor oxygen and water transport, CNC increases the crystalline regions, due to the hydrogen bonding network formed, and leads to edible films with higher stability and lower permeability [65,66,67].

On the other hand, due to its hydrophobic nature, CD provides a high moisture barrier and improves water resistance [54]. Thus, the positive effect on the barrier properties is attributed to both the hydrophobic nature of CD and the forces between CD and CH or HPMC that enhance the stability of the composite films.

Upon incorporation of AgNPs, the OP and WVP values decreased significantly (*p* < 0.05) for both types of composite film (for 15% *v*/*v* incorporation of AgNPs, in CH-CNC-CD films the reduction of OP was by 40% and of WVP by 58%, while in HPMC-CNC-CD films it was by 38% for OP and by 57% for WVP), being in agreement with relevant studies on the effect of nanoparticles on barrier properties [66,68,69,70]. More specifically, AgNPs seem to act as a discontinuous barrier against oxygen and water vapor diffusion, which increases the path length of diffusion. As in the case of CNC, the increase in crystalline regions, with the formation of a hydrogen bonding network, leads to higher stability and improved barrier properties [65,66]. In any case, the addition of 15% *v*/*v* AgNPs to both types of films provided the best overall results for these properties.

#### 3.1.3. Optical Properties

Color difference from a white plate (ΔE), white index (WI) and chrome (C*) of the film samples are presented in Table 3.

The optical properties are of key importance, as they certainly influence the consumers’ preference and the product demand. According to the results in Table 3, the HPMC-CNC-CD films, before incorporating AgNPs, showed a lower ΔΕ value than CH-CNC-CD (30.37 vs. 47.59), a lower WI value (60.87% vs. 65.79%) and the same C* value (1.41). Both films were quite transparent, as indicated by the combination of high WI values with low C* values [71]. The results in the case of HPMC-CNC-CD films are indeed consistent with the fact that the HPMC solutions were highly transparent [50,72]. On the other hand, the CH-CNC-CD films were light yellow as the base material (CH) was yellow in color, whereas HPMC was white.

The fact that both CH and HPMC films present quite satisfactory optical properties was also indicated by the experiments described in Bizymis et al. [28,29], which showed that 1% *w*/*v* HPMC plain films were slightly superior to 1% *w*/*v* CH plain films in their ΔE and WI properties (31.99 and 63.06% in HPMC films vs. 36.17 and 58.87% in CH films respectively), while CH was slightly superior in C* (1.61 in CH films vs. 2.23 in HPMC films).

With the addition of CNC and CD, the properties of both types of composite film remained at satisfactory levels, as it is shown in Table 3. This can be attributed to the fine dispersion of CNC and CD in the CH and HPMC matrices [73].

Upon incorporation of AgNPs, the C* values decreased (for 15% *v*/*v* incorporation, to 0.86 in CH-CNC-CD films and 0.42 in HPMC-CNC-CD films), resulting in a lower color intensity, that is desirable for edible films [43]. The other two properties (ΔΕ and WI) were enhanced in the case of CH-CNC-CD (for 15% *v*/*v* incorporation, to 31.69 and 69.78%, respectively) and remained at the same level in the case of HPMC-CNC-CD. Overall, by dispersing in the film matrices, AgNPs contributed to higher transparency and homogeneity for both types of films [48]. In any case, the addition of 15% *v*/*v* AgNPs to both types of films provided the best overall results for these properties.

#### 3.1.4. Antimicrobial Activity

Table 4 presents the % reduction of microbial growth and the antimicrobial activity factor (R) of the edible films against *E. coli*, which is a food-borne pathogenic bacterium.

**Figure 1:**
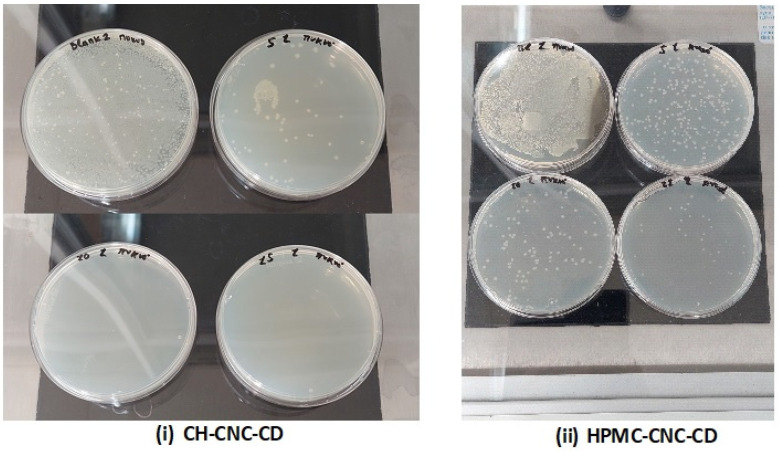
Growth of *E. coli* colonies in Petri dishes for 0% *v*/*v* (top left), 5% *v*/*v* (top right), 10% *v*/*v* (bottom left) and 15% *v*/*v* (bottom right) AgNPs, with use of undiluted SCDLP in all cases.

According to JIS L 1902:2015 [47] and Pittol et al. [74], to be considered effective, R must be equal to or higher than 2.0. As expected, films without AgNPs did not show any antimicrobial activity, but with the addition of AgNPs, the antimicrobial activity became strong for both types of films (Table 4). Especially, in almost all cases, the R value was higher than three, that means a very good antimicrobial activity. The only exception was in the case of CH-CNC-CD films with 5% *v*/*v* AgNPs, where the R value was lower than 2 (1.40), representing a medium antimicrobial activity. However, the reduction of microbial growth was still quite high (96.01%). The higher performance of HPMC-CNC-CD films incorporating 5% *v*/*v* AgNPs, compared to that of the corresponding CH-CNC-CD films, is probably related to the better dispersion of AgNPs in the polymer matrices. However, in the case of CH-CNC-CD films with 10% *v*/*v* AgNPs, the R factor reached a value of 4.50, with the reduction of microbial growth being 99.99%. These results are also illustrated in Figure 1, which shows the growth of *E. coli* colonies in Petri dishes, using undiluted SCDLP in all cases. For both types of films, the addition of 15% *v*/*v* AgNPs gave the highest R values, as expected.

The antimicrobial activity of AgNPs depends on their shape and size. AgNPs are effective antimicrobial agents because of their crystallographic surface structure and large surface-to-volume ratios [23,24]. More specifically, the cell wall of *E. coli* has a complex structure with a thin layer of peptidoglycan surrounded by an outer membrane. AgNPs can lead to the formation of many cavities and gaps and the accumulation of nanoparticles in the bacterial membrane and the cytoplasmic regions of cells. Due to the production of oxygen reactive species and the accumulation of AgNPs, the bacterial cells are destroyed. Furthermore, the AgNPs can penetrate inside cells and disturb their metabolism [13,24,75].

These results are in agreement with relevant studies which support that AgNPs can provide antimicrobial activity to edible films [13,23,24,75,76,77]. However, the quantitative study of antimicrobial activity by calculating the R factor is still at an early stage, as mainly qualitative studies have been reported so far. However, comparing the current results with other research results, it can be argued that the current material combinations give higher R values. For example, Fontecha-Umaña et al. [77] created polyester films with higher concentrations of AgNPs (400, 500, 650 and 850 ppm) and calculated values of 2.35, 4.14, 4.67 and 4.90 for the R factor, respectively, whereas, in the current study, even lower concentrations of AgNPs led to higher R factors, especially in the case of HPMC-CNC-CD films.

### 3.2. Investigation of the Application of the Formed Edible Films to Cherries

Cherries are highly appreciated by consumers for their special flavor. However, their quality deteriorates rapidly after their harvesting. The main characteristics of cherry deterioration are color changes, softening, surface opening and an increase in microbial load. Edible films and coatings offer a way to reduce all these defects and maintain the quality of cherries at a high quality during their storage time. This paragraph examines the use of CH-CNC-CD and HPMC-CNC-CD edible films for cherries, on the one hand, without AgNPs and, on the other hand, with AgNP additions of 5% *v*/*v* and 15% *v*/*v*. During storage time, the properties studied at regular intervals were weight loss (WL), color difference (ΔE), hardness and total microbial load.

#### 3.2.1. Weight Loss (WL)

**Figure 2:**
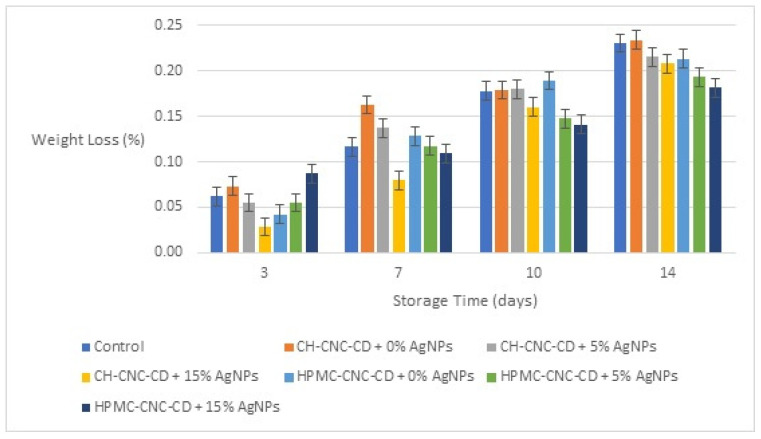
Change in weight loss (WL) of cherries during storage for all the samples.

The evaluation of weight loss (WL) for all types of cherry samples started from the 3rd day of storage, as shown in Figure 2 (obviously there was no weight loss on 0 day). As can be seen, during storage, WL increases. However, WL values generally remained low due to the protection afforded to the cherries by their peel. In comparison, however, the samples coated with the edible films where AgNPs were added suffered lower WL in most cases, and especially on the 14th day of storage. This result can be explained by the reduced values of OP and WVP for edible films with AgNPs, as described in Section 3.1.2. When films with low OP and low WVP are applied to fruit and vegetables, they can prevent moisture loss and the ingress of external moisture and microorganisms, thus ensuring their freshness and extending their shelf life [75]. Moreover, the samples with HPMC-CNC-CD coatings generally showed lower WL values than those with CH-CNC-CD, which is consistent with the lower OP and WVP values of HPMC-CNC-CD films compared to those with CH-CNC-CD.

#### 3.2.2. Color Difference (ΔE)

**Figure 3:**
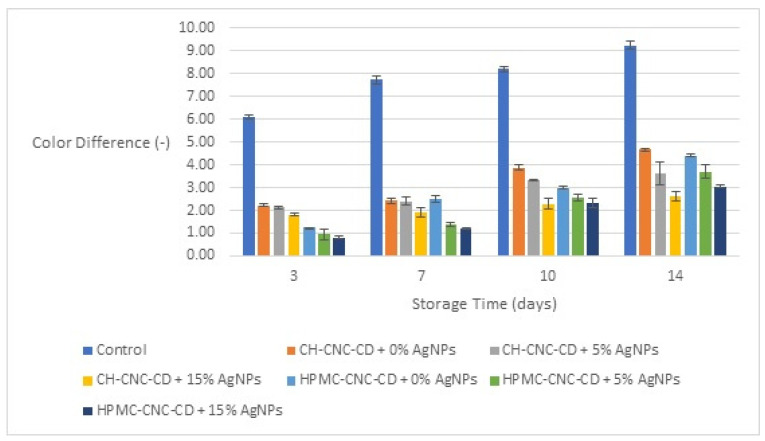
Change in color difference (ΔE) of cherries during storage for all the samples.

The evaluation of the color difference (ΔE) for all types of cherry samples started from the 3rd day of storage, as shown in Figure 3, in order to ensure that the coating is fully stabilized and the results are representative. As observed, the use of edible films is very important since, at all times, the control samples had at least 5 units more ΔE than those with the edible films. Also, samples with AgNPs showed the lowest values (lower than four in all cases, even on the 14th day), which can be explained by the advanced transparency of the edible films with AgNPs and the reduced interactions with the environment, leading to lower color changes. Finally, the type of film (CH-CNC-CD or HPMC-CNC-CD) had no significant effect on the results for this property.

#### 3.2.3. Hardness

**Figure 4:**
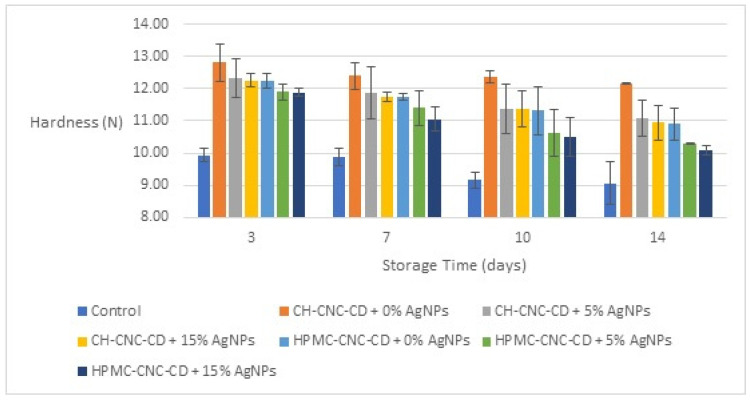
Change in hardness of cherries during storage for all the samples.

On 0 day, the hardness of the control samples was measured at 9.957 N. The hardness evaluation for all types of cherry samples started from the 3rd day of storage, as shown in Figure 4, in order to ensure that the coating is fully stabilized and the results are representative. The results show that the samples coated with edible films had a higher hardness, which is obviously important as it can reduce the surface opening during storage and keep the food quality at high levels for a longer time. It is possible that the films applied, and especially those with CH as a base material, made the skin of the cherries harder, due to their stability and high mechanical properties. The addition of AgNPs led to lower hardness values compared to the coated samples without AgNPs, but to higher values compared to the control samples.

#### 3.2.4. Total Microbial Load (log CFU/g)

**Figure 5:**
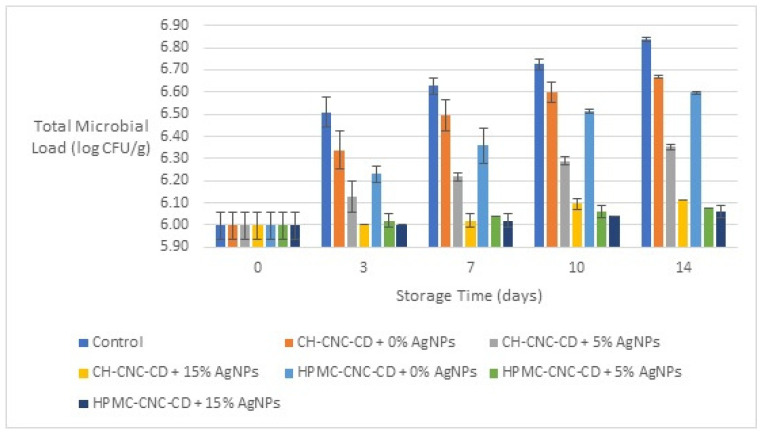
Change in total microbial load of cherries during storage for all the samples.

The results for the total microbial load (log CFU/g) of all samples during the storage period are shown in Figure 5. From the results, the importance of the edible films, and especially of the films with the addition of AgNPs, is evident, as all the coated samples showed significantly better results than the control samples (e.g., on 14th day, with incorporation of 5% *v*/*v* AgNPs, the total microbial load was 6.35 log CFU/g in the CH-CNC-CD coated samples and 6.08 log CFU/g in the HPMC-CNC-CD coated samples, while, with incorporation of 15% *v*/*v* AgNPs, it was 6.11 log CFU/g and 6.06 log CFU/g, respectively, against 6.84 log CFU/g of the control samples). These results can be attributed to the low permeability of the coated surface of cherries and mainly to the antimicrobial activity of the AgNPs. Finally, samples with the HPMC-CNC-CD coatings showed a lower total microbial load than those with CH-CNC-CD, which can also be explained by the lower permeability values of HPMC-CNC-CD films compared to those of CH-CNC-CD.

#### 3.2.5. Statistical Analysis of the Results for the Cherry Samples

Due to the large number of parameters (time, application or not of coating, choice of base material, cases of AgNPs incorporation), the comparative presentation of the changes of properties over time for all parameters simultaneously was considered more appropriate to display in bar charts.

Table 5 presents a statistical analysis of the results according to the criteria of time and type of sample (control or coated).

As shown in Table 5, the properties change in a statistically significant manner over time in almost all cases. The only case that did not show a statistically significant change was the total microbial load from the 10th day to the 14th day. However, it must be noted that, after the 14 days of storage, the coated samples, and especially those with incorporation of AgNPs, were preserved in a good condition.

On the other hand, the type of sample (control sample or with each of the six different coatings) causes statistically significant changes to the properties in a large number of cases.

In particular, the property values of the control samples are statistically significantly different from the property values of all the coated samples, except for the weight loss values of the CH-CNC-CD + 5% AgNPs and the HPMC-CNC-CD + 0% AgNP samples, for which there was no statistically significant difference. However, it should be taken into account that, as already mentioned, the weight loss values generally remained low during the storage period.

Regarding the coated samples, the most statistically significant differences between the samples (four different groups) occurred in the total microbial load, while there were three different groups in the weight loss and the hardness and two different groups in the color difference.

## 4. Conclusions

The results of the current study point out that AgNPs contributed significantly to the antimicrobial activity of CH-CNC-CD and HPMC-CNC-CD edible films, while they additionally led to lower thickness and improved barrier and optical properties in both films. Indicatively, it is stated that, by 15% *v*/*v* AgNP incorporation into a film, the thickness reduced by 0.03 mm in the CH-CNC-CD film and by 0.02 mm in the HPMC-CNC-CD film; the OP and the WVP reduced by 40% and 58%, respectively, in the CH-CNC-CD film, and by 38% and 57%, respectively, in the HPMC-CNC-CD film; the color intensity C* reduced by 39% in the CH-CNC-CD film and by 70% in the HPMC-CNC-CD film. Finally, by 10% *v*/*v* AgNP incorporation, the reduction of microbial growth reached 99.99% in both films.

The above edible films/coatings proved effective in the preservation of fresh fruit cherries, improving their properties such as color difference and total microbial load. Finally, on the 14th day of storage, by 5% *v*/*v* AgNP incorporation in the film, the total microbial load was 6.35 log CFU/g in the CH-CNC-CD coated fruits and 6.08 log CFU/g in the HPMC-CNC-CD coated fruits, while by 15% *v*/*v* AgNPs incorporation it was 6.11 log CFU/g and 6.06 log CFU/g, respectively, against 6.84 log CFU/g of the control samples.

Further work is needed to investigate the safety issues of the proposed use of edible films/coatings with AgNPs in fruit preservation, such as the updated regulatory limitations in relation to nanoparticles, and/or the possibility of AgNP removal by washing just before consumption, as well as the potential impact of AgNPs on sensory properties of the coated fruits.

## Figures and Tables

**Table 1:** Thickness and mechanical properties of the formed edible films.

Sample	AgNPs (% *v*/*v*)	Thickness (mm)	F (N)	Elastic Modulus (N/mm)	ε (%)	σ (MPa)
CH-CNC-CD	0	0.04 ± 0.00 ^aA^	19.31 ± 1.13 ^aA^	5.76 ± 0.47 ^aA^	16.78 ± 0.39 ^aA^	0.98 ± 0.06 ^aA^
	5	0.02 ± 0.00 ^aB^	19.66 ± 0.01 ^aA^	4.94 ± 0.09 ^aA^	19.90 ± 0.35 ^aA^	1.00 ± 0.00 ^aA^
	10	0.02 ± 0.00 ^aB^	19.63 ± 0.02 ^aA^	4.59 ± 0.12 ^aA^	21.38 ± 0.53 ^aA^	1.00 ± 0.00 ^aA^
	15	0.01 ± 0.01 ^aB^	17.91 ± 0.11 ^aA^	4.34 ± 0.05 ^aA^	20.65 ± 0.35 ^aA^	0.91 ± 0.01 ^aA^
HPMC-CNC-CD	0	0.03 ± 0.00 ^bA^	13.44 ± 2.39 ^bA^	4.75 ± 0.38 ^aA^	14.08 ± 1.38 ^bA^	0.68 ± 0.12 ^bA^
	5	0.01 ± 0.00 ^bB^	12.94 ± 2.33 ^bA^	4.18 ± 0.41 ^aA^	15.40 ± 1.27 ^bA^	0.66 ± 0.12 ^bA^
	10	0.01 ± 0.00 ^bB^	16.72 ± 2.75 ^bA^	4.68 ± 0.51 ^aA^	17.80 ± 0.99 ^bA^	0.85 ± 0.14 ^bA^
	15	0.01 ± 0.00 ^bB^	12.40 ± 3.77 ^bA^	4.10 ± 0.44 ^aA^	14.98 ± 3.01 ^bA^	0.63 ± 0.19 ^bA^

Values are given as mean ± standard deviation. Different letters in the same column indicate significant differences (*p* < 0.05) based on Duncan’s test difference criterion. Small letters indicate differences based on the basic film material (CH or HPMC) while capital letters show differences based on the quantity of AgNPs.

**Table 2:** Barrier properties of the formed edible films.

Sample	AgNPs (% *v*/*v*)	OP (g·s^−1^·Pa^−1^·m^−1^) · 10^−12^	WVP (g·s^−1^·Pa^−1^·m^−1^) · 10^−9^
CH-CNC-CD	0	1.89 ± 0.09 ^aA^	2.26 ± 0.08 ^aA^
	5	1.11 ± 0.01 ^aB^	1.52 ± 0.00 ^aB^
	10	1.12 ± 0.01 ^aB^	1.57 ± 0.00 ^aB^
	15	1.14 ± 0.01 ^aB^	1.40 ± 0.00 ^aB^
HPMC-CNC-CD	0	1.60 ± 0.03 ^bA^	1.72 ± 0.00 ^bA^
	5	0.54 ± 0.01 ^bB^	0.72 ± 0.00 ^bB^
	10	0.55 ± 0.01 ^bB^	0.74 ± 0.00 ^bB^
	15	0.57 ± 0.00 ^bB^	0.74 ± 0.00 ^bB^

Values are given as mean ± standard deviation. Different letters in the same column indicate significant differences (*p* < 0.05) based on Duncan’s test difference criterion. Small letters indicate differences based on the basic film material (CH or HPMC) while capital letters show differences based on the quantity of AgNPs.

**Table 3:** Optical properties of the formed edible films.

Sample	AgNPs (% *v*/*v*)	ΔE (-)	WI (%)	C* (-)
CH-CNC-CD	0	47.59 ± 2.86 ^aA^	65.79 ± 1.69 ^aA^	1.41 ± 0.69 ^aA^
	5	32.15 ± 0.03 ^aA^	69.01 ± 0.03 ^aA^	1.24 ± 0.02 ^aA^
	10	31.33 ± 0.02 ^aA^	69.93 ± 0.02 ^aA^	1.08 ± 0.01 ^aA^
	15	31.69 ± 0.05 ^aA^	69.78 ± 0.04 ^aA^	0.86 ± 0.08 ^aA^
HPMC-CNC-CD	0	30.37 ± 0.19 ^aA^	60.87 ± 0.21 ^aA^	1.41 ± 0.15 ^aA^
	5	30.73 ± 0.12 ^aA^	60.27 ± 0.12 ^aA^	0.54 ± 0.02 ^aA^
	10	31.22 ± 0.11 ^aA^	59.74 ± 0.11 ^aA^	0.51 ± 0.03 ^aA^
	15	30.03 ± 0.05 ^aA^	60.92 ± 0.05 ^aA^	0.42 ± 0.01 ^aA^

Values are given as mean ± standard deviation. Different letters in the same column indicate significant differences (*p* < 0.05) when analyzed by the Duncan’s Test. Small letters indicate differences based on the basic film material (CH or HPMC) while capital letters show differences based on the quantity of AgNPs.

**Table 4:** Antimicrobial activity of the formed edible films.

Sample	AgNPs (% *v*/*v*)	R (-)	% Reduction of Microbial Growth
CH-CNC-CD	0	0.00 ± 0.00 ^aA^	0.00 ± 0.00 ^aA^
	5	1.40 ± 0.03 ^aB^	96.01 ± 0.29 ^aB^
	10	4.50 ± 0.00 ^aB^	99.99 ± 0.01 ^aB^
	15	4.50 ± 0.00 ^aB^	99.99 ± 0.01 ^aB^
HPMC-CNC-CD	0	0.00 ± 0.00 ^bA^	0.00 ± 0.00 ^aA^
	5	6.76 ± 0.15 ^bB^	99.99 ± 0.01 ^aB^
	10	6.92 ± 0.14 ^bB^	99.99 ± 0.01 ^aB^
	15	7.56 ± 0.07 ^bB^	99.99 ± 0.01 ^aB^

Values are given as mean ± standard deviation. Different letters in the same column indicate significant differences (*p* < 0.05) when analyzed by the Duncan’s Test. Small letters indicate differences based on the basic film material (CH or HPMC) while capital letters show differences based on the quantity of AgNPs.

**Table 5:** Results with statistical analysis for coated cherries samples.

Storage Time (Days)	Sample	WL (%)	ΔE (-)	Hardness (N)	Total Microbial Load (log CFU/g)
3	Control	0.06 ± 0.01 ^aA^	6.09 ± 0.09 ^aA^	9.930 ± 0.202 ^aA^	6.51 ± 0.07 ^aA^
	CH-CNC-CD + 0% AgNPs	0.07 ± 0.01 ^bA^	2.23 ± 0.04 ^bA^	12.820 ± 0.578 ^bA^	6.34 ± 0.08 ^bA^
	CH-CNC-CD + 5% AgNPs	0.06 ± 0.01 ^aA^	2.13 ± 0.06 ^bA^	12.325 ± 0.591 ^cA^	6.13 ± 0.07 ^cA^
	CH-CNC-CD + 15% AgNPs	0.03 ± 0.01 ^cA^	1.81 ± 0.06 ^cA^	12.258 ± 0.199 ^cA^	6.00 ± 0.00 ^dA^
	HPMC-CNC-CD + 0% AgNPs	0.04 ± 0.01 ^aA^	1.24 ± 0.02 ^bA^	12.237 ± 0.224 ^cA^	6.23 ± 0.04 ^eA^
	HPMC-CNC-CD + 5% AgNPs	0.06 ± 0.01 ^cA^	0.94 ± 0.23 ^cA^	11.898 ± 0.259 ^dA^	6.02 ± 0.03 ^dA^
	HPMC-CNC-CD + 15% AgNPs	0.09 ± 0.01 ^cA^	0.81 ± 0.07 ^cA^	11.891 ± 0.137 ^dA^	6.00 ± 0.00 ^dA^
7	Control	0.12 ± 0.01 ^aB^	7.72 ± 0.16 ^aB^	9.886 ± 0.269 ^aB^	6.63 ± 0.04 ^aB^
	CH-CNC-CD + 0% AgNPs	0.16 ± 0.01 ^bB^	2.42 ± 0.12 ^bB^	12.406 ± 0.414 ^bB^	6.50 ± 0.07 ^bB^
	CH-CNC-CD + 5% AgNPs	0.14 ± 0.01 ^aB^	2.41 ± 0.15 ^bB^	11.872 ± 0.798 ^cB^	6.22 ± 0.02 ^cB^
	CH-CNC-CD + 15% AgNPs	0.08 ± 0.01 ^cB^	1.90 ± 0.21 ^cB^	11.747 ± 0.125 ^cB^	6.02 ± 0.03 ^dB^
	HPMC-CNC-CD + 0% AgNPs	0.13 ± 0.01 ^aB^	2.52 ± 0.16 ^bB^	11.736 ± 0.101 ^cB^	6.36 ± 0.08 ^eB^
	HPMC-CNC-CD + 5% AgNPs	0.13 ± 0.01 ^cB^	1.39 ± 0.08 ^cB^	11.399 ± 0.530 ^dB^	6.04 ± 0.00 ^dB^
	HPMC-CNC-CD + 15% AgNPs	0.11 ± 0.01 ^cB^	1.19 ± 0.03 ^cB^	11.055 ± 0.368 ^dB^	6.02 ± 0.03 ^dB^
10	Control	0.18 ± 0.01 ^aC^	8.19 ± 0.12 ^aC^	9.163 ± 0.238 ^aC^	6.72 ± 0.02 ^aC^
	CH-CNC-CD + 0% AgNPs	0.18 ± 0.01 ^bC^	3.88 ± 0.11 ^bC^	12.360 ± 0.188 ^bC^	6.60 ± 0.05 ^bC^
	CH-CNC-CD + 5% AgNPs	0.18 ± 0.01 ^aC^	3.32 ± 0.05 ^bC^	11.373 ± 0.766 ^cC^	6.29 ± 0.02 ^cC^
	CH-CNC-CD + 15% AgNPs	0.16 ± 0.01 ^cC^	2.29 ± 0.25 ^cC^	11.376 ± 0.564 ^cC^	6.10 ± 0.02 ^dC^
	HPMC-CNC-CD + 0% AgNPs	0.19 ± 0.01 ^aC^	3.00 ± 0.08 ^bC^	11.327 ± 0.744 ^cC^	6.51 ± 0.01 ^eC^
	HPMC-CNC-CD + 5% AgNPs	0.15 ± 0.01 ^cC^	2.54 ± 0.15 ^cC^	10.637 ± 0.721 ^dC^	6.06 ± 0.03 ^dC^
	HPMC-CNC-CD + 15% AgNPs	0.14 ± 0.01 ^cC^	2.31 ± 0.19 ^cC^	10.493 ± 0.606 ^dC^	6.04 ± 0.00 ^dC^
14	Control	0.23 ± 0.01 ^aD^	9.24 ± 0.15 ^aD^	9.061 ± 0.657 ^aD^	6.84 ± 0.01 ^aC^
	CH-CNC-CD + 0% AgNPs	0.23 ± 0.01 ^bD^	4.66 ± 0.06 ^bD^	12.147 ± 0.019 ^bD^	6.67 ± 0.01 ^bC^
	CH-CNC-CD + 5% AgNPs	0.22 ± 0.01 ^aD^	3.62 ± 0.49 ^bD^	11.091 ± 0.569 ^cD^	6.35 ± 0.01 ^cC^
	CH-CNC-CD + 15% AgNPs	0.21 ± 0.01 ^cD^	2.61 ± 0.22 ^cD^	10.942 ± 0.540 ^cD^	6.11 ± 0.00 ^dC^
	HPMC-CNC-CD + 0% AgNPs	0.21 ± 0.01 ^aD^	4.39 ± 0.06 ^bD^	10.907 ± 0.491 ^cD^	6.60 ± 0.01 ^eC^
	HPMC-CNC-CD + 5% AgNPs	0.19 ± 0.01 ^cD^	3.70 ± 0.29 ^cD^	10.288 ± 0.028 ^dD^	6.08 ± 0.00 ^dC^
	HPMC-CNC-CD + 15% AgNPs	0.18 ± 0.01 ^cD^	3.04 ± 0.07 ^cD^	10.090 ± 0.138 ^dD^	6.06 ± 0.03 ^dC^

Values are given as mean ± standard deviation. Different letters in the same column indicate significant differences (*p* < 0.05) based on Duncan’s test difference criterion. Small letters indicate differences based on the type of the cherries’ samples (control or coated) while capital letters show differences based on the storage time.

## Data Availability

All data generated or analyzed during this study are included in this published article.

## Abbreviations

AFM	atomic force microscopy
AgNPs	silver nanoparticles
C*	chrome
CD	beta-cyclodextrin
CH	chitosan
CNC	cellulose nanocrystals
F	maximum breaking force
HPMC	hydroxypropyl methylcellulose
OP	oxygen permeability
R	antimicrobial activity
WI	whiteness index
WL	weight loss
WVP	water vapor permeability
XRD	X-ray diffraction
ΔE	color difference
ε	elongation
σ	breaking stress
